# Measurement of Thermal Effects of Doppler Ultrasound: An *In Vitro* Study

**DOI:** 10.1371/journal.pone.0135717

**Published:** 2015-08-24

**Authors:** Samir Helmy, Yvonne Bader, Marianne Koch, Denise Tiringer, Christian Kollmann

**Affiliations:** 1 Department of Obstetrics and Gynecology, Medical University Vienna, Vienna, Austria; 2 Department of Gynecology, Obstetrics and Reproductive Medicine, University Clinic of Saarland, Homburg, Germany; 3 Karl Landsteiner Society, Austria; 4 Center for Medical Physics and Biomedical Engineering, Medical University Vienna, Vienna, Austria; University of Illinois at Urbana-Champaign, UNITED STATES

## Abstract

**Objective:**

Ultrasound is considered a safe imaging modality and is routinely applied during early pregnancy. However, reservations are expressed concerning the application of Doppler ultrasound in early pregnancy due to energy emission of the ultrasound probe and its conversion to heat. The objective of this study was to evaluate the thermal effects of emitted Doppler ultrasound of different ultrasound machines and probes by means of temperature increase of in-vitro test-media.

**Methods:**

We investigated the energy-output of 5 vaginal and abdominal probes of 3 ultrasound machines (GE Healthcare, Siemens, Aloka). Two in-vitro test objects were developed at the Center for Medical Physics and Biomedical Engineering, Medical University Vienna (water bath and hydrogel bath). Temperature increase during Doppler ultrasound emission was measured via thermal sensors, which were placed inside the test objects or on the probes’ surface. Each probe was emitting for 5 minutes into the absorbing test object with 3 different TI/MI settings in Spectral Doppler mode.

**Results:**

During water bath test, temperature increase varied between 0.1 and 1.0°C, depending on probe, setting and focus, and was found highest for spectral Doppler mode alone. Maximum temperature increase was found during the surface heating test, where values up to 2.4°C could be measured within 5 minutes of emission.

**Conclusions:**

Activation of Doppler ultrasound in the waterbath model causes a significant increase of temperature within one minute. Thermally induced effects on the embryo cannot be excluded when using Doppler ultrasound in early pregnancy.

## Introduction

Ultrasound is generally considered a safe imaging modality in obstetrics. [1–3] However, when ultrasound travels through tissue, energy is absorbed by the tissue components and converted to heat dependent on frequency and intensity.[4] These thermal effects may alter the equilibrium between chemical reactions and therefore may consequently harm the surrounding tissue. [5–10] The official joint statement by the World Federation of Ultrasound in Medicine and Biology (WFUMB) and the International Society of Ultrasound in Obstetrics and Gynecology (ISUOG) on the use of Doppler pregnancy in early pregnancy states that the use of medical ultrasound in obstetrics is safe for B and M modes, but reservations are expressed for Doppler, especially spectral and colour Doppler. [11–13] Also the American Institute of Ultrasound in Medicine (AIUM) states reservations regarding the use of ultrasound in early pregnancy. Even though no congenital anomalies could be clearly associated with ultrasound use, bioeffects may be more subtle and/or have a low incidence. As these effects are difficult to detect with epidemiologic studies, the AIUM stresses on the importance of further laboratory studies to clearly identify physical mechanisms of ultrasound in early pregnancy. [14]

Consequently education of ultrasound operators stresses, that pulsed wave Doppler (PW-Doppler) examination produces high-energy acoustic outputs and should not be used in the early first trimester of pregnancy due to the risk of potential damage to the fragile embryo. [15–18] Therefore doppler ultrasound should only be performed in case of a valid medical indication, and the lowest possible ultrasonic exposure setting should be used. [2,9,16,19]

In 1993 the American Federal Drugs Administration (FDA) raised the upper spatial-peak temporal-average (I_SPTA_) limit of commercially available ultrasound devices from 94mW/cm^2^ to 720 mW/cm^2^. Since that time all Ultrasound-monitors have to display two safety indices. The thermal index (TI) is the ratio of the power used to that required to produce a temperature rise of 1°C. A higher TI value indicates a higher heating potential of the US probe and subsequently a higher actual risk for tissue damage, however, a TI of 1 does not automatically imply a temperature increase of 1°C at any location within the ultrasound field.[14]

There are three sub-indices, the thermal index in soft tissue (TIS), the thermal index in bone (TIB), and the thermal index in the cranium (TIC). TIS assumes that the ultrasound only reaches soft tissue, like during ultrasound in the first trimester, whereas TIB should be appropriately displayed during second and third trimester. TIC assumes that the transducer is very close to bone and applies in neonatal, pediatric and adult patients. The second index that has to be displayed is the mechanical index (MI), which is intended to offer a guide to the likelihood of a nonthermal bioeffect including cavitation and is related to the intensity of the pulse. [11,20–22]

The BMUS safety guidelines state that a MI over 0.3 may increase the risk of capillary bleeding, and a MI higher than 0.7 may cause cavitation. Therefore it is advised to limit the MI under 0,7 when embryonic tissue is scanned.

If subliminal physical changes are happening during scanning at increased intensity levels, potential effects may not become apparent. In this in- vitro study we aimed to investigate the thermal effects of different ultrasound machines and the resulting change of temperature in the tissue surrounding the probe. Simulating a realistic setting in which the ultrasound user is not trained in specific sub- settings of the ultrasound machine (TIS/TIB/TIC), we wanted to demonstrate the maximum energy output one can reach when using different ultrasound probes (vaginal and abdominal) at different settings.

## Material and Methods

Three different ultrasound machines, which were all available at the Department of Obstetrics and Gynaecology (Medical University of Vienna), were used for the conduction of this experiment: GE Voluson E8, Siemens Versa Pro, and Aloka Prosound alpha 7.

Of these ultrasound machines we investigated both vaginal and abdominal probes. (Table 1)

**Table 1:** Investigated ultrasound machines and vaginal/ abdominal probes.

Ultrasound machine	Probe
GE Voluson E8	IC 5-9D (2D vaginal)
	RIC 6-12D (vaginal)
	RAB 4-8D (3D abdominal)
Siemens Versa Pro	6,5 EV13
	3,5 C40
Aloka Prosound alpha 7	TV 4–8 (vaginal)
	TV 3D (vaginal)
	CV 4–8

For the in-vitro tests two special test objects were developed at the Center for Medical Physics and Biomedical Engineering (Medical University of Vienna).

**Fig 1:**
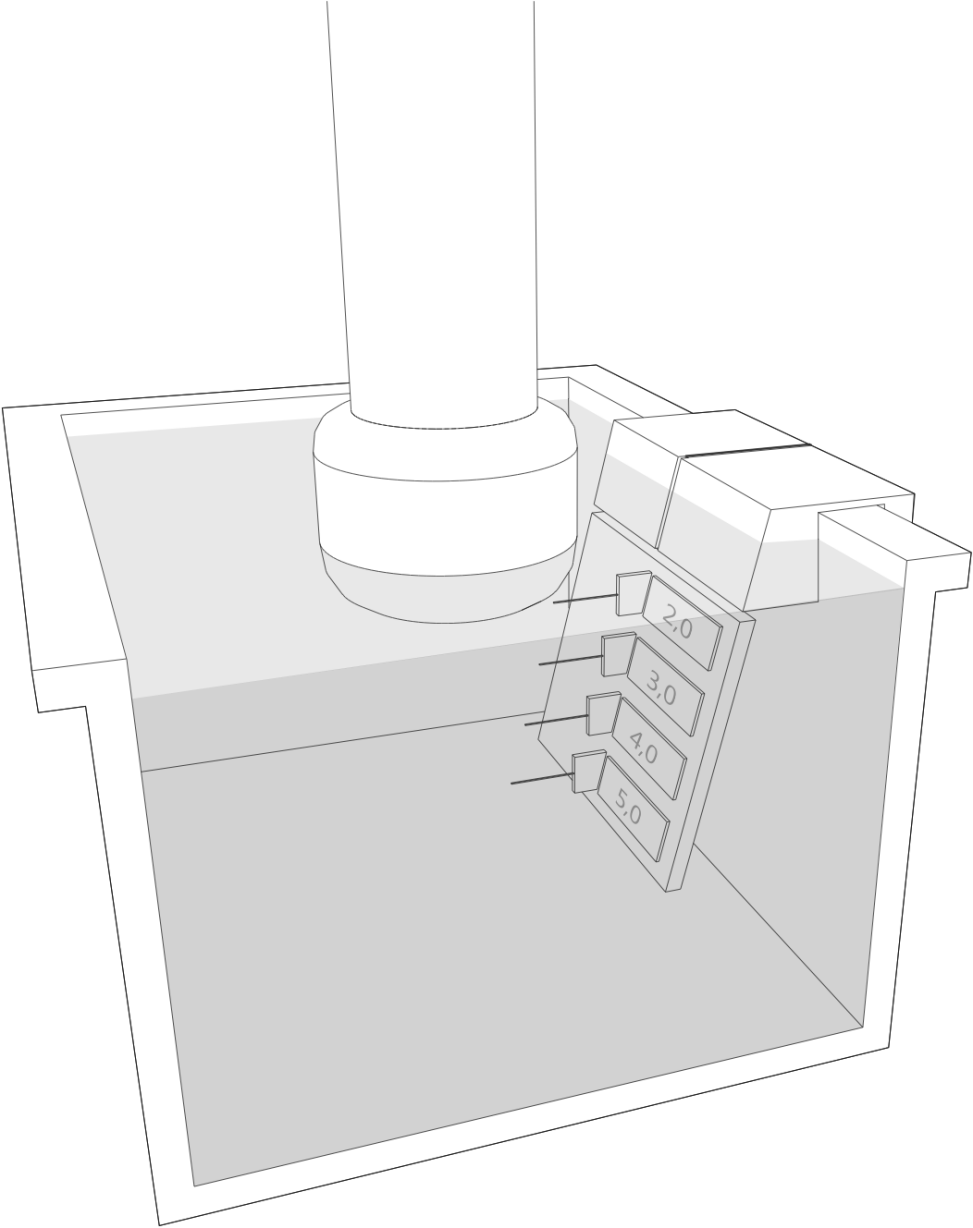
Waterbath test object. Water- filled persplex cube, which contained 4 thermal micro sensors (Betatherm Ltd.) in 4 different equidistant depths (2–5 cm). For each testing, the specific probe was placed at the surface of the water bath object in a manner that all thermal sensors were within the ultrasound beam.

One test object was a water- filled persplex cube, which contained 4 thermal micro sensors (Betatherm Ltd.) in 4 different equidistant depths (2–5 cm). For each testing, the specific probe was placed at the surface of the water bath object in a manner that all thermal sensors were within the ultrasound beam (Fig 1)In order to prevent multi-reflexions or standing waves, the bottom of the water- filled test object was covered with a high effective damping material (Aptflex, Precision Acoustics, UK).

The thermal sensors had a diameter of 0.46 mm and a length of the active area of 3.18 mm with a rapid thermal response time of 200 ms. All sensors were connected via a multiplexer and an A/D-converter (10 bit, National Instruments) to a PC. The data from the sensors (sampling rate 5 Hz) were visualized and stored with a custom-made acquisition program using DASYLab 1992–2013 (National Instruments Ireland Resources Limited). During the developing process the sensors were calibrated over a temperature range of 15–50°C with an overall tolerance of ± 0.2°C.

During pretests, the settings of each ultrasound machine system were changed to obtain the maximum of the displayed Thermal Index (TI) value available with each probe and console.

These pretests were done in order to simulate reality in which any user of the ultrasound device can change the settings individually. The resulting set-ups were then documented together with the displayed TI and Mechanical Index (MI) values and used for the following measurements.

After positioning of the non-emitting probe on the test object, the sample volume and the focus were directed to the depth of the second sensor (at 3 cm).

This was done to show that the heating of the sensor was not influenced by the probes’ surface, but only by the settings of the probe. This was confirmed as the sensor closest to the probe (2 cm), did not reach the temperature as the second sensor down below (3 cm).

The Doppler path was arranged in a way that all thermal sensors (2–5 cm) were touched from that virtual line.

The ultrasound system was then unfrozen and the heating profile was measured over 1 minute for all sensors. This process was repeated 2 times after a cooling down phase of 5 minutes.

**Fig 2:**
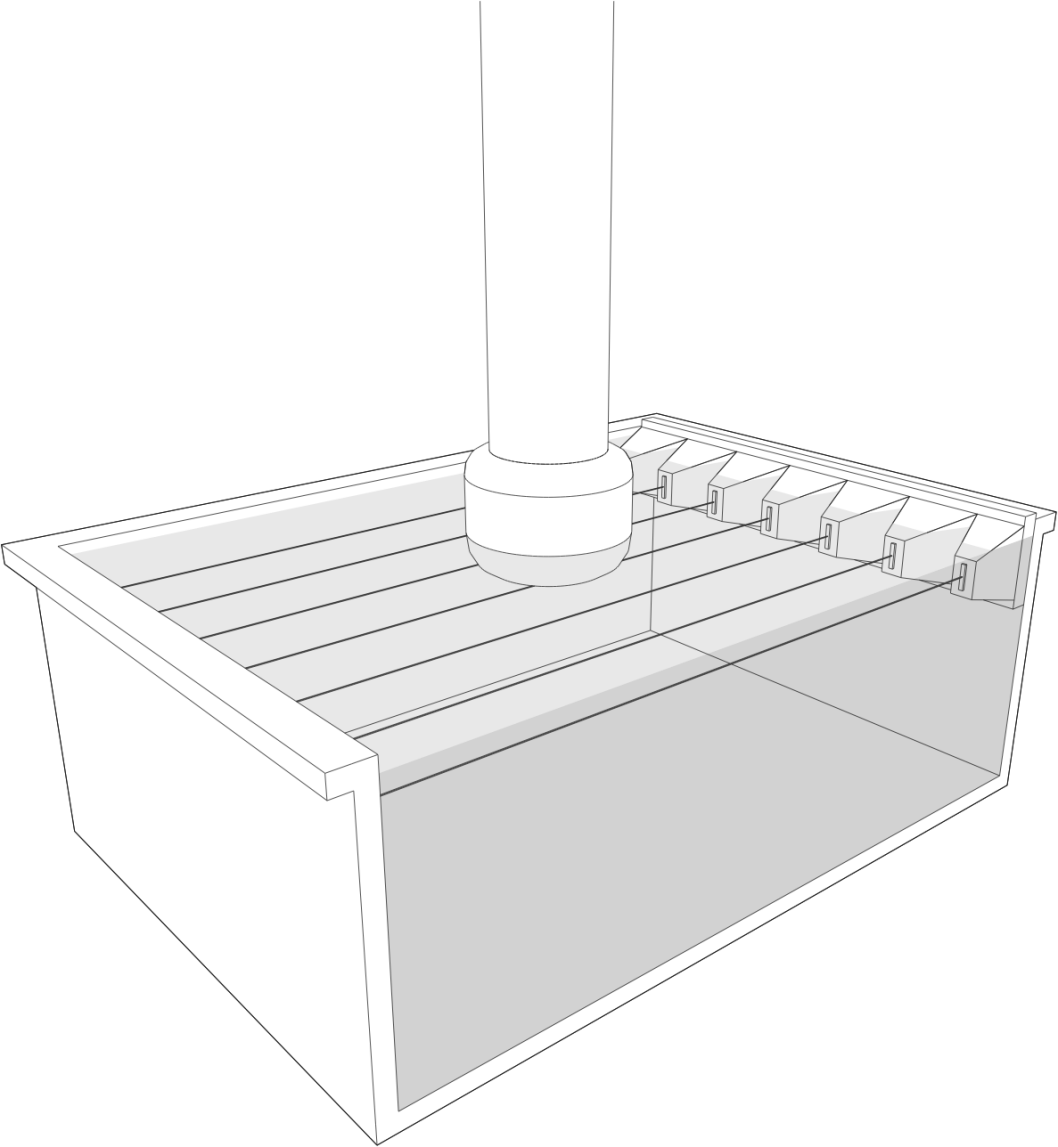
Surface heating test object. Persplex cube containing hydrogel; sides covered with damping material (Aptflex, Precision Acoustics, UK). Surface heating of the different probes was measured via a sensor, which was attached to the probe’s surface within the Doppler sound path.

For the surface heating measurements, a second test object was developed similar to the first one, but containing hydrogel instead of water. All sides and the bottom of the persplex cube were covered with the same damping material (Aptflex, Precision Acoustics, UK). At the probe´s surface specifically the region, which contains the active elements for the chosen mode is heating up very quickly.The surface heating of the different probes was measured via a sensor, which was attached to the probe’s surface within the Doppler sound path (Fig 2).

In total we investigated the surface heating of 5 different probes of 2 systems (TV 4–8, CV 4–8: Aloka; RAB 4–8 ABD, IC 5-9D, RIC 6–12: GE).

During the experiment, each probe was emitting for 5 minutes into the absorbing test object with 3 different TI/MI settings in Spectral Doppler mode and Transcranial Doppler program to allow highest system output values.

Institutional Review Board (IRB) approval was not required for this study.

## Results

### Water bath measurements

The results of all measurements and probes show a variation of 0,1 to 1.0°C, dependent on probe, setting and focus.

Table 2 shows the maximal temperature increases after 1 min with special TI/MI settings in the water bath model.

**Table 2:** Water bath model. Maximal temperature increase after 1 minute at different TI/ MI settings.

Probe	Max.TI (TIS/TIB/TIC)	MI	Mode	Max. temp. Increase at 1 min (°C)
IC5-9D (GE)	-/-/1.0	0.2	SD + CD	+0.1
	-/-/0.7	-	SD+CD	+ 0.1
	-/-/0.8	0.4	SD	+0.2–0.3
	-/-/0.7	0.2	SD	+0.5
RIC-12D (GE)	0.2/-/-	0.1	SD	+0.1 (Foc S1)
	0.4/-/-	0.1	SD	+0.1 (Foc S2)
	0.2/-/-	0.6	SD+CD	0.0
RAB 4-8D (GE)	0.2/-/-	0.6	SD	+0.3 (Foc S3)
	0.4/-/-	0.7	SD	+0.2 (Foc S2)
	0.2/-/-	1.2	SD+CD	+0.1 (Foc S2)
	-/-/0.6	0.4	SD	+0.2 (Foc S2)
	-/-/0.6	0.2	SD+CD	+0.1
6,5EV13 (Siemens)	0.6/2.0/-	0.6	SD	+0.6 (Foc S2)
3,5C40 (Siemens)	1.0/3.5/-	< 0.4	SD	+0.5 (Foc S2)
	1.5/4.5/-	0.6	SD	+0.5 (foc S3)
	1.5/-/-	-	SD	+0.3 (Foc S3)
	-/3.5/-	-	SD	+1.0 (Foc S3)
	-/3.5/-	-	SD	+0.8 (Foc S4)
TV 4–8 (Aloka)	<0.4/-/-	1.1	SD	+0.1 (Foc S2)
	-/0.8/-	0.75	SD	+0.2 (Foc S2)
	-/0.8/-	0.75	SD+CD	+0.2 (Foc S2)
TV-3D (Aloka)	-/2.2/-	0.95	SD+CD	+0.4(FocS2)
	-/1.4/-	1,2	SD+CD	+0.3 (FocS2)
	-/2.4/-	1.1	SD+CD	+0.4 (focS2)
Convex Sector (UST-9115-5) (Aloka)	-/2.2/-	0.85	SD+CD	+0.2 (FocS2)
	-/1.0/-	0.65	SD+CD	+0.1 (FocS2)

TI is displayed as the TI setting, at which the highest temperature increase could be measured (e.g. probe IC5-9D: TIC setting showed a larger increase in temperature in all measurements compared to TIS or TIB); (TIS) Thermal Index Soft- Tissue (TIB) Thermal Index Bone (TIC) Thermal Index Cranium; Spectral Mode (SD), Colour Mode (CD)

### Surface heating measurements

We could measure values up to 2.4°C increase of temperature within five minutes of emission. (Table 3) We could observe a trend, that probes with an active element count of 145 to 192 showed maximal temperature increases in the surface heating test.

**Table 3:** Surface heating of probes within 5 minutes of spectral- Doppler and color Doppler emission.

Probe	TIB	MI	Mode	temp. increase within 5 min [°C]
TV 4–8 (Aloka)	0.6	0.61	SD	+ 1.5
	0.8	0.61	SD	+ 1.7
	1.0	0.73	SD	+ 2.4
TV 4–8 (Aloka)	0.8	0.63	SD+CD	+ 2.4(4–5 mm away): + 0.9
CV 4–8 (Aloka)	<0.4	0.73	SD+CD	+ 1.2
RAB 4–8 ABD (GE)	TIC = 0.7	0.4	SD+CD	+ 1.4
	TIC = 0.1	0.3	SD+CD	+0.6
	TIC = 0.6	0.3	SD+CD	+ 1.1
IC 5-9D (GE)	TI = 0.5	0.3	SD+CD	+0.6
RIC 6–12 (GE)	TI = 0.2	0.7	SD+CD	+2.5

In total, five of the investigated probes (Aloka and Siemens) exceeded the recommended threshold levels of TI for routine ultrasound investigation, which is given by the British Medical Ultrasound Society (BMUS). [23]

The highest temperature increases were observed in the surface heating tests, whereas lower increases were shown in the water bath test.

## Discussion

The temperature increase depends on the special modes chosen (color (CD), Spectral (SD), combination) and is slightly highest for spectral Doppler mode alone. It furthermore depends on the focal and sample volume positions within the field: if both positions coincided and a sensor was within the position, a maximum temperature increase could be measured. The sensors above or below measured lower increases. Furthermore, probes with increasing number of active elements showed a maximum MI change, whereas it remains unclear whether the number of active elements influences the TI.

Our results showed that measured temperature rise and TI are not directly related. The TI is a calculated value, which allows only a rough estimation of temperature increase. For the user of an ultrasound system it might therefore be hard to estimate where the highest temperature increases could occur within the sound field. Additionally the temperature increase varies from system settings and probe used.

In the course of this study, we focused on the TI, but did not investigate the MI. Therefore, our results are solemnly related to the measurement of temperature rise via temperature receptors, but not to the mechanical effects that are caused by the intensity of the pulse released from the ultrasound probe.

However, temperature rise caused by Doppler ultrasound may produce a biothermal effect on tissue.

Epidemiological studies in the human population have shown, although controversial, that there might be an association between ultrasound exposure in pregnancy and some traits such as left handedness, lower birth weight and delayed speech. [24,25] A recent study in mice furthermore suggested a potential correlation to autism, as pups, which were exposed to intra- uterine ultrasound showed significantly less interest in social interaction compared to the sham group. [26]

Thermally induced bioeffects of ultrasound, which may even lead to teratogenesis, have already been suggested in a variety of studies, including in vitro and animal studies. [8,10,16,24,27–31]

In the results section we could demonstrate the highest temperature increase during the surface heating test. This finding might be specifically relevant for the use of vaginal probes during early pregnancy, as the probes’ surface is placed at a close localization to the embryo.

One of the limitations of this study is that due to ethical reasons, it is not justifiable to test thermic effects by Doppler ultrasound on human live embryos- we are therefore dependent on assumptions resulting from animal studies and theoretical research models. Therefore, a weakness of our study is that we cannot directly draw a clinical conclusion from this in- vitro study on early pregnancy effects of Doppler ultrasound. In our in-vitro study we experimented with different TI settings, and also TI settings, which are not supposed to be used in early pregnancy scans, in order to demonstrate highest possible increases in heat. Ultrasound scanning is routinely done in early pregnancy, however in reality not all ultrasound examiners may be skilled in the use of ultrasound machines and may therefore accidentally work with TI settings which are not appropriate in early pregnancy.

On the other hand, our experimental setting could not consider the specific maternal and fetal in-vivo physiology. Produced heat by the ultrasound probe may immediately be dissipated through maternal and/or fetal blood circulation. We also did not reproduce the layers of tissue normally separating the ultrasound probe from the early pregnancy, including amniotic fluid. However, in our water bath measurement we could show that heating of the sensor was not influenced by the probes’ surface, but only by the settings of the probe. The sensor on which the focus was directed reached a higher temperature than the sensor closer to the probes’ surface. These results may suggest that barriers of tissue between the probe and the early pregnancy may be irrelevant when the focus is set on the fetus.

One strength of the current study is that the in- vitro experiment was conducted in a cooperative setting between the Department of Gynecology and the Department of Medical Physics, which therefore includes a clinical as well as technical perspective.

We conclude that guidelines on the safe use of Doppler ultrasound should be closely followed as Doppler may increase temperature of surrounding tissue, which could have unkown effects to the embryo. Our in-vitro experiment showed that at activation of Doppler ultrasound, temperature in situ was rising up to 1°C within one minute during the waterbath test. Therefore Doppler ultrasound in early pregnancy should only be applied when needed and within the shortest time possible With this study we can confirm the BMUS consensus paper on the safe use of Doppler ultrasound in early pregnancy.

## Supporting Information

S1 File Raw data for water bath measurement and surface heating measurement. (ZIP)

## References

[1] Merritt CR (1989) Ultrasound safety: what are the issues? Radiology 173: 304–306. 2678243 10.1148/radiology.173.2.2678243

[2] Abramowicz JS (2013) Benefits and risks of ultrasound in pregnancy. Semin Perinatol 37: 295–300. 10.1053/j.semperi.2013.06.004 24176149

[3] Torloni MR, Vedmedovska N, Merialdi M, Betran AP, Allen T, et al (2009) Safety of ultrasonography in pregnancy: WHO systematic review of the literature and meta-analysis. Ultrasound Obstet Gynecol 33: 599–608. 10.1002/uog.6328 19291813

[4] O'Brien WD Jr. (2007) Ultrasound-biophysics mechanisms. Prog Biophys Mol Biol 93: 212–255. 16934858 10.1016/j.pbiomolbio.2006.07.010 PMC1995002

[5] Hoskins P MK, Trush A, Whittingham TA (2003) Diagnostic Ultrasound; Physics and Equipment. London Greenwich Medical.

[6] Meizner I (2012) [What do doctors understand regarding ultrasound safety during pregnancy?]. Harefuah 151: 234–236, 252 22616153

[7] Browne JE, Ramnarine KV, Watson AJ, Hoskins PR (2003) Assessment of the acoustic properties of common tissue-mimicking test phantoms. Ultrasound Med Biol 29: 1053–1060. 12878252 10.1016/s0301-5629(03)00053-x

[8] Church CC, Miller MW (2007) Quantification of risk from fetal exposure to diagnostic ultrasound. Prog Biophys Mol Biol 93: 331–353. 16949653 10.1016/j.pbiomolbio.2006.07.015

[9] Houston LE, Odibo AO, Macones GA (2009) The safety of obstetrical ultrasound: a review. Prenat Diagn 29: 1204–1212. 10.1002/pd.2392 19899071

[10] (1999) Thermal teratology. European Committee for Medical Ultrasound Safety (ECMUS). Eur J Ultrasound 9: 281–283. 10657603

[11] Society SGotBMU (2009) Guidelines for the safe use of diagnostic ultrasound equipment. In: Society TBMU, editor.

[12] Hershkovitz R, Sheiner E, Mazor M (2002) Ultrasound in obstetrics: a review of safety. Eur J Obstet Gynecol Reprod Biol 101: 15–18. 11803093 10.1016/s0301-2115(01)00469-9

[13] Wfumb/Isuog (2013) WFUMB/ISUOG statement on the safe use of Doppler ultrasound during 11–14 week scans (or earlier in pregnancy). Ultrasound Med Biol 39: 373 10.1016/j.ultrasmedbio.2012.11.025 23398714

[14] Fowlkes JB, Bioeffects Committee of the American Institute of Ultrasound in M (2008) American Institute of Ultrasound in Medicine consensus report on potential bioeffects of diagnostic ultrasound: executive summary. J Ultrasound Med 27: 503–515. 18359906 10.7863/jum.2008.27.4.503

[15] Abramowicz JS, Kossoff G, Marsal K, Ter Haar G, International Society of Ultrasound in O, et al (2003) Safety Statement, 2000 (reconfirmed 2003). International Society of Ultrasound in Obstetrics and Gynecology (ISUOG). Ultrasound Obstet Gynecol 21: 100 12528176 10.1002/uog.36

[16] Bly S, Van den Hof MC, Diagnostic Imaging Committee SoO, Gynaecologists of C (2005) Obstetric ultrasound biological effects and safety. J Obstet Gynaecol Can 27: 572–580. 16100635 10.1016/s1701-2163(16)30716-2

[17] Barnett SB, Maulik D, International Perinatal Doppler S (2001) Guidelines and recommendations for safe use of Doppler ultrasound in perinatal applications. J Matern Fetal Med 10: 75–84. 11392597 10.1080/714904312

[18] Henderson J, Willson K, Jago JR, Whittingham TA (1995) A survey of the acoustic outputs of diagnostic ultrasound equipment in current clinical use. Ultrasound Med Biol 21: 699–705. 8525560 10.1016/0301-5629(94)00158-a

[19] ter Haar GR, Abramowicz JS, Akiyama I, Evans DH, Ziskin MC, et al (2013) Do we need to restrict the use of Doppler ultrasound in the first trimester of pregnancy? Ultrasound Med Biol 39: 374–380. 10.1016/j.ultrasmedbio.2012.11.024 23332816

[20] Kollmann C, ter Haar G, Dolezal L, Hennerici M, Salvesen KA, et al (2013) Ultrasound emissions: thermal and mechanical indices. Ultraschall Med 34: 422–431; quiz 432–424. 10.1055/s-0033-1335843 23860856

[21] Kollmann C (2007) New sonographic techniques for harmonic imaging—underlying physical principles. Eur J Radiol 64: 164–172. 17875378 10.1016/j.ejrad.2007.07.024

[22] Sheiner E, Abramowicz JS (2012) A symposium on obstetrical ultrasound: is all this safe for the fetus? Clin Obstet Gynecol 55: 188–198. 10.1097/GRF.0b013e3182488386 22343238

[23] Society) TBMUSSGotBMU (2003) Guidelines for the safe use of diagnostic ultrasound equipment.

[24] Edwards MJ, Shiota K, Smith MS, Walsh DA (1995) Hyperthermia and birth defects. Reprod Toxicol 9: 411–425. 8563185 10.1016/0890-6238(95)00043-a

[25] Salvesen KA (2002) EFSUMB: safety tutorial: epidemiology of diagnostic ultrasound exposure during pregnancy-European committee for medical ultrasound safety (ECMUS). Eur J Ultrasound 15: 165–171. 12423743 10.1016/s0929-8266(02)00038-1

[26] McClintic AM, King BH, Webb SJ, Mourad PD (2014) Mice exposed to diagnostic ultrasound in utero are less social and more active in social situations relative to controls. Autism Res 7: 295–304. 10.1002/aur.1349 24249575 PMC4025980

[27] Abramowicz JS, Barnett SB, Duck FA, Edmonds PD, Hynynen KH, et al (2008) Fetal thermal effects of diagnostic ultrasound. J Ultrasound Med 27: 541–559; quiz 560–543. 18359908 10.7863/jum.2008.27.4.541

[28] Horder MM, Barnett SB, Vella GJ, Edwards MJ, Wood AK (1998) In vivo heating of the guinea-pig fetal brain by pulsed ultrasound and estimates of thermal index. Ultrasound Med Biol 24: 1467–1474. 10385968 10.1016/s0301-5629(98)00111-2

[29] Pfaffenberger S, Vyskocil E, Kollmann C, Unger E, Kaun C, et al (2013) Transtemporal ultrasound application potentially elevates brain temperature: results of an anthropomorphic skull model. Ultraschall Med 34: 51–57. 10.1055/s-0032-1313083 22872379

[30] Vyskocil E, Pfaffenberger S, Kollmann C, Gleiss A, Nawratil G, et al (2012) Thermal Effects of Diagnostic Ultrasound in an Anthropomorphic Skull Model. Ultraschall Med. 10.1055/s-0032-1312924 22744443

[31] Jensh RP, Brent RL (1999) Intrauterine effects of ultrasound: animal studies. Teratology 59: 240–251. 10331527 10.1002/(SICI)1096-9926(199904)59:4<240::AID-TERA10>3.0.CO;2-V

