# Supplementary figures and images for: Measurement of Thermal Effects of Doppler Ultrasound: An In Vitro Study
Source: PLoS One. 2015 Aug 24;10(8):e0135717. doi: 10.1371/journal.pone.0135717 (PMC4547707; doi:10.1371/journal.pone.0135717)

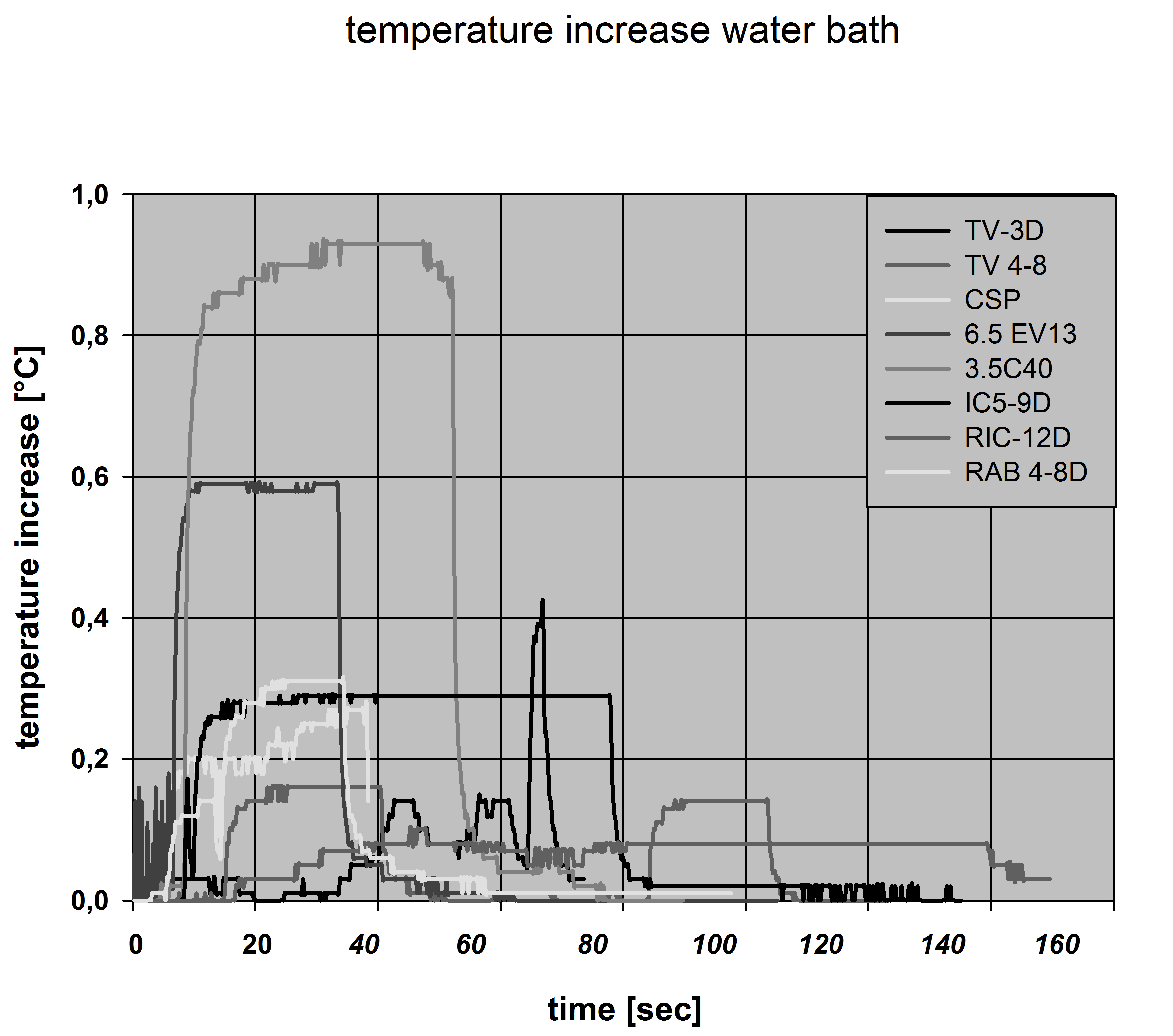

Supplement: S1 File — (ZIP) [file pone.0135717.s001.zip › rawData_4PlosOne/surface/fig3_greyscale_lines.JPG]

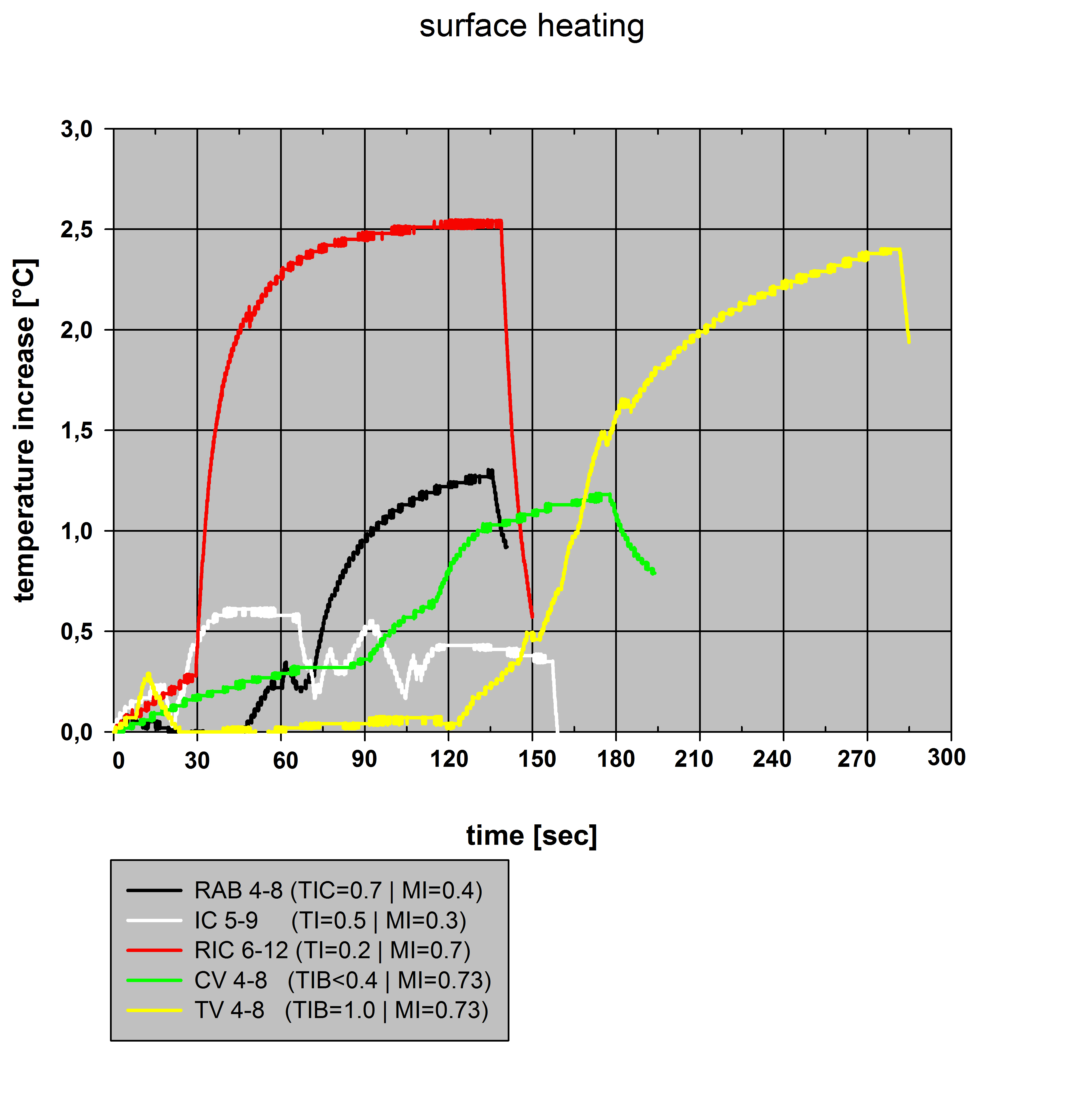

Supplement: S1 File — (ZIP) [file pone.0135717.s001.zip › rawData_4PlosOne/surface/surfaceHeating_1.jpg]

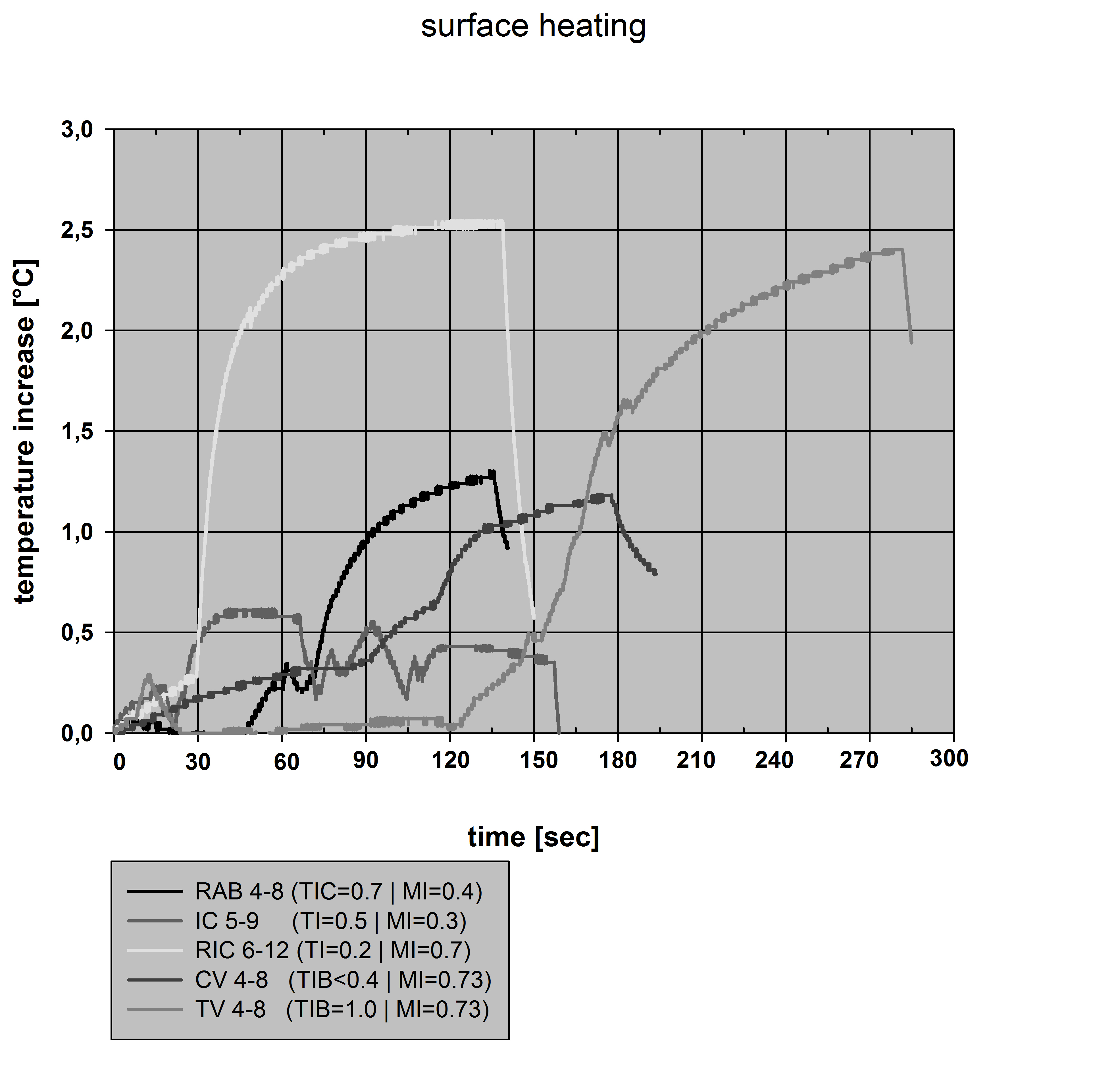

Supplement: S1 File — (ZIP) [file pone.0135717.s001.zip › rawData_4PlosOne/surface/surfaceHeating_1_greyscale_lines.jpg]

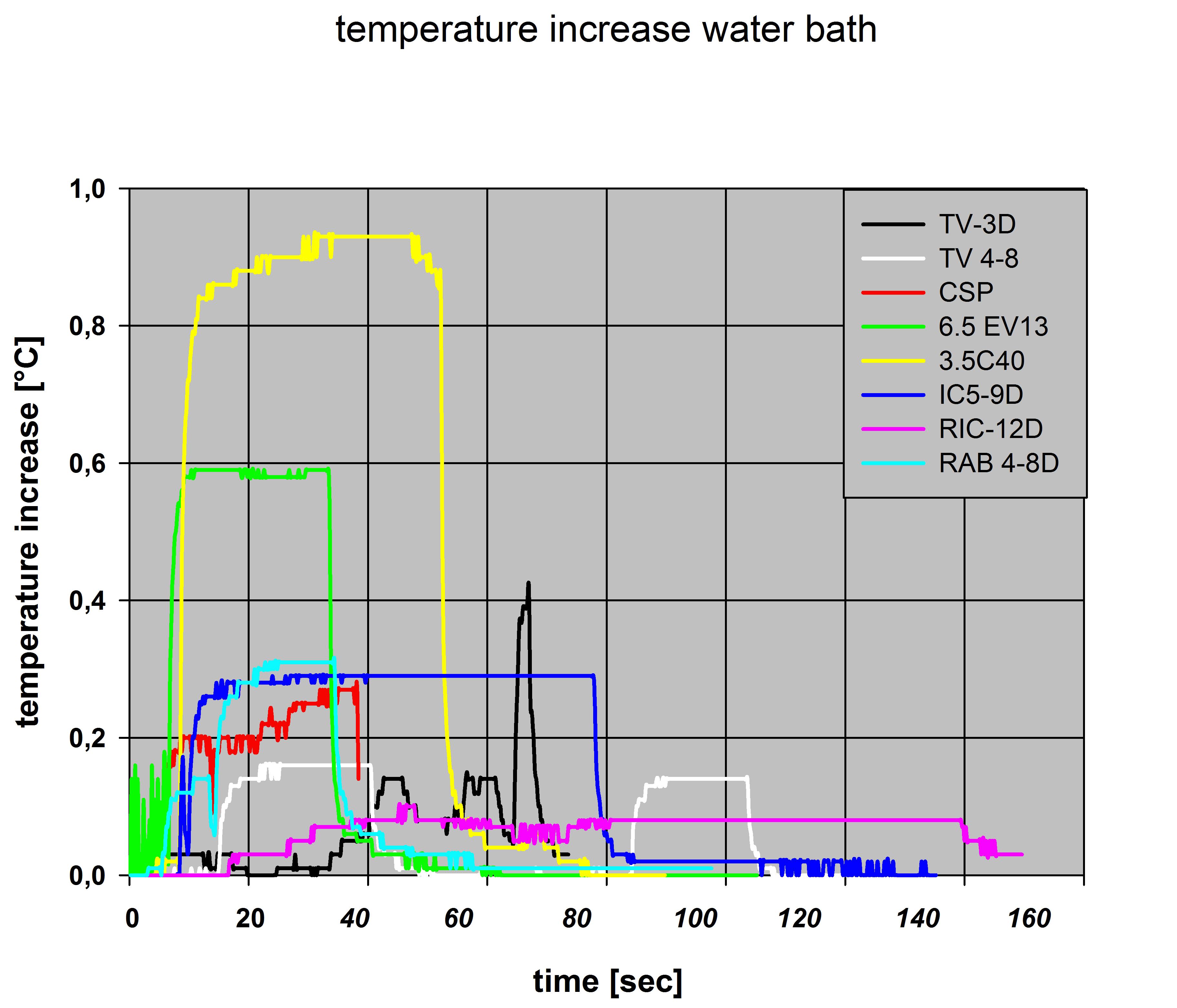

Supplement: S1 File — (ZIP) [file pone.0135717.s001.zip › rawData_4PlosOne/water/temp_waterbath.jpg]
